# Supplementary material for: Hippocampus as a sorter and reverberatory integrator of sensory inputs
Source: Nat Commun. 2022 Dec 20;13:7413. doi: 10.1038/s41467-022-35119-2 (PMC9768143; doi:10.1038/s41467-022-35119-2)
Supplement: Supplementary file 1 — Supplementary Information [file 41467_2022_35119_MOESM1_ESM.pdf]

Supplementary Information for

Hippocampus as a sorter and reverberatory integrator of sensory inputs

Masanori Nomoto[1,2,3], Emi Murayama[1,2,3], Shuntaro Ohno[1,2,3], Reiko Okubo-Suzuki[1,2,3], Shin-ichi Muramatsu[4,5], Kaoru Inokuchi[1,2,3,*]

[1]Research Centre for Idling Brain Science, University of Toyama; Toyama 930-0194, Japan.

[2]Department of Biochemistry, Graduate School of Medicine and Pharmaceutical Sciences, University of Toyama; Toyama 930-0194, Japan.

[3]CREST, JST, University of Toyama; Toyama 930-0194, Japan.

[4]Division of Neurology, Department of Medicine, Jichi Medical University; Tochigi 329-0498, Japan.

[5]Center for Gene and Cell Therapy, The Institute of Medical Science, The University of Tokyo; Tokyo 108-8639, Japan.

*Corresponding author. Email: inokuchi@med.u-toyama.ac.jp

Supplementary Figures

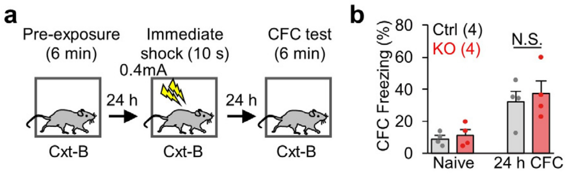

Supplementary Figure. 1 CA3-NR1 KO mice exhibited comparable contextual freezing in the pre-exposure-facilitated CFC task.

a Experimental design. **b** Contextual freezing levels in 24 h long-term memory test. *P* values were calculated using an unpaired two-tailed *t* test. N.S., not significant ($P > 0.05$). Graphs represent means ± SEM, and circles in the graph represent individual animals. Numbers in parentheses denote the number of mice in each group used for the study. Lightning bolt, footshock; Cxt, context; HPC, hippocampus; AP, anterior-posterior; N.S., not significant.

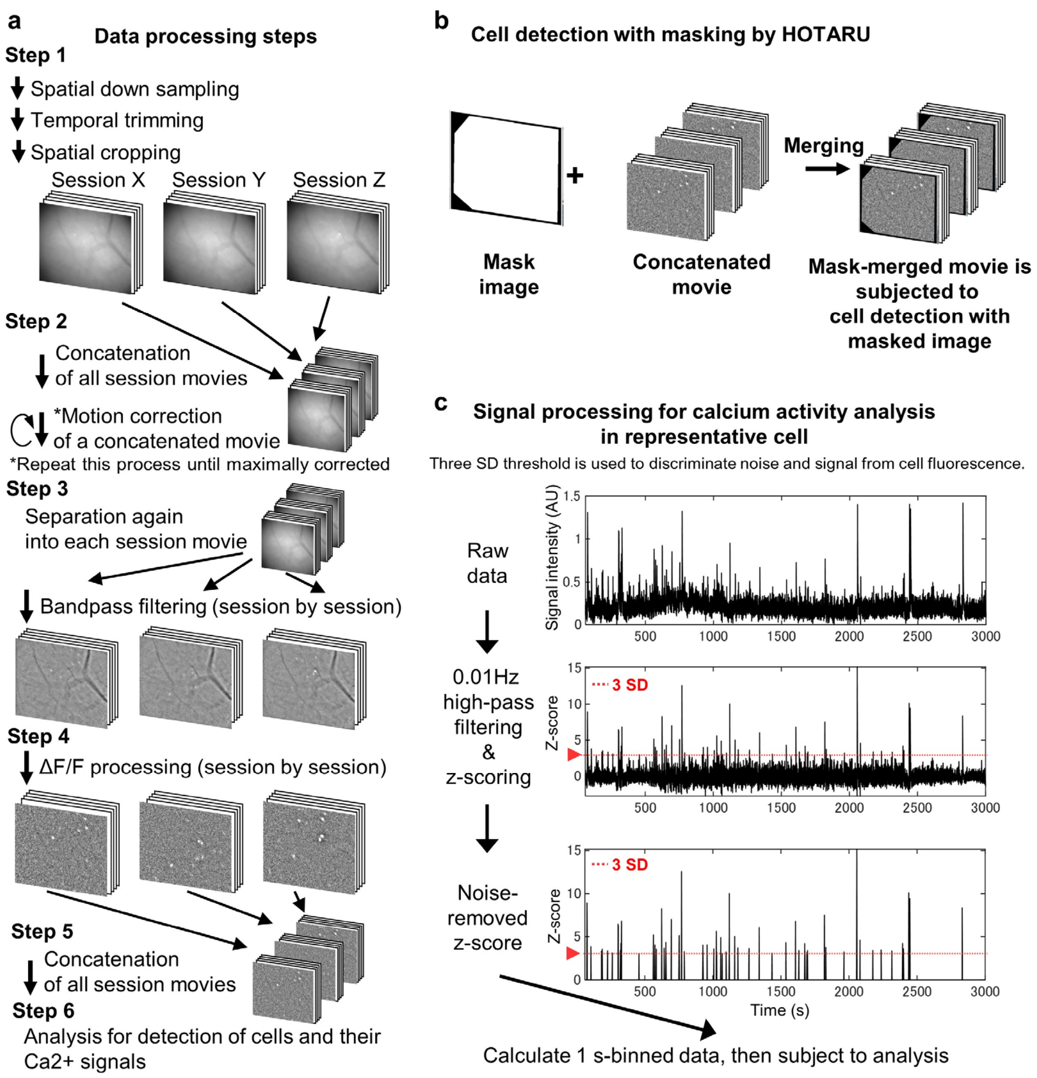

Supplementary Figure. 2 Data processing and cell detection for analysis

a Data processing steps. Step 1: movies acquired from each behavioral session are down-sampled, temporally trimmed, and spatially cropped. Step 2: the movies are concatenated into a series of movies, and repeatedly corrected until artifacts of movement are minimized. Step 3: the concatenated movie is separated again into pre-concatenated movies and subsequently subjected to bandpass filtering. Step 4: dF/F conversion. Step 5: re-concatenation. Step 6: the concatenated- and registrated-movie is subjected to cell detection. **b** A train of dF/F is subjected into the HOTARU algorithm to automatically detect active cells. **c** The acquired calcium trace signal is converted into calcium activity by high-pass filtering, z-score normalization, and cutoff of inadequate signal in each cell to remove background fluctuation.

Then 1 s-mean calcium activities are subjected to quantitative analyses. The dashed line indicates a cutoff threshold less than three standard deviations (< 3 SD).

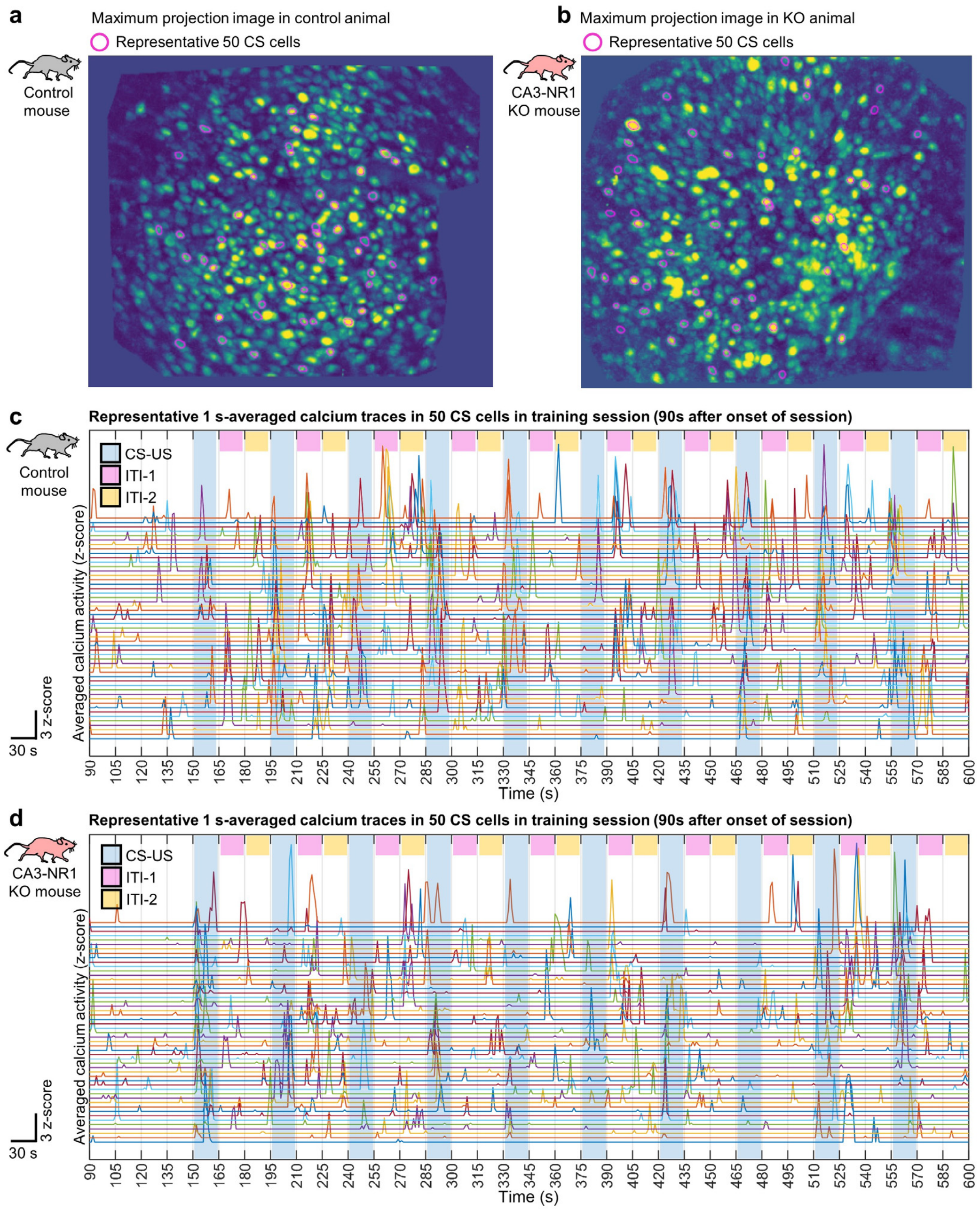

Supplementary Figure. 3 Representative *in vivo* calcium imaging data acquisition in CA1 of freely moving animals

a, **b** Stacked- and pseudo-colored dF/F images acquired using the microendoscope over entire recording sessions of imaging in the hippocampus from (**a**) control and (**b**) KO animals. Magenta circles indicate the footprint contours of detected cells. **c**, **d** Representative 1 s-averaged calcium activities in representative 50 CS-responsive cells during LFC training 90 s after onset of behavioral session to end in (**c**) control and (**d**) KO animals. Blue, pink, and yellow rectangles indicate the timings of CS-US, ITI-1, and ITI-2, respectively.

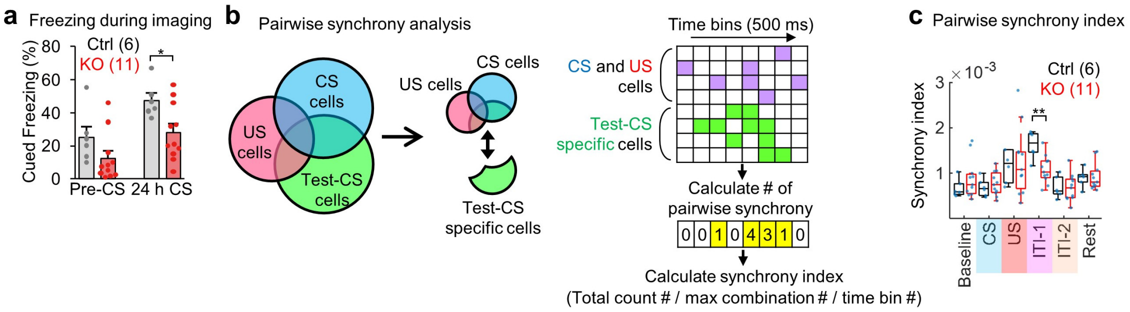

Supplementary Figure. 4 CA3-NR1 KO mice exhibited impaired functional synchrony between CS ∪ US- and Test-CS-responsive specific cells.

a Cued freezing levels during 24 h long-term memory tests in the imaging study (two-tailed unpaired Student's t test, *P* = 0.023). **b** Scheme for synchrony analysis. Binarized $Ca2^+$ activity in each cell is sorted into CS ∪ US- and Test-CS-responsive specific subpopulations, and then pairwise synchrony is calculated by normalizing the number of synchronizations every 500 ms in each session. **c** Box plots comparing the mean synchrony between genotypes in each session (two-tailed unpaired Student's t test, *P* = 0.003). Numbers in parentheses denote the number of mice (**a**, **c**) in each group used for the study. *P* values were calculated using an unpaired two-tailed *t* test (**a**, **c**) ($*P < 0.05$, $**P < 0.01$). Box plots illustrate median, first, and third quantiles, and minimum and maximum values. Graphs and scatter plots represent means ± SEM. In graphs, circles represent individual animals.

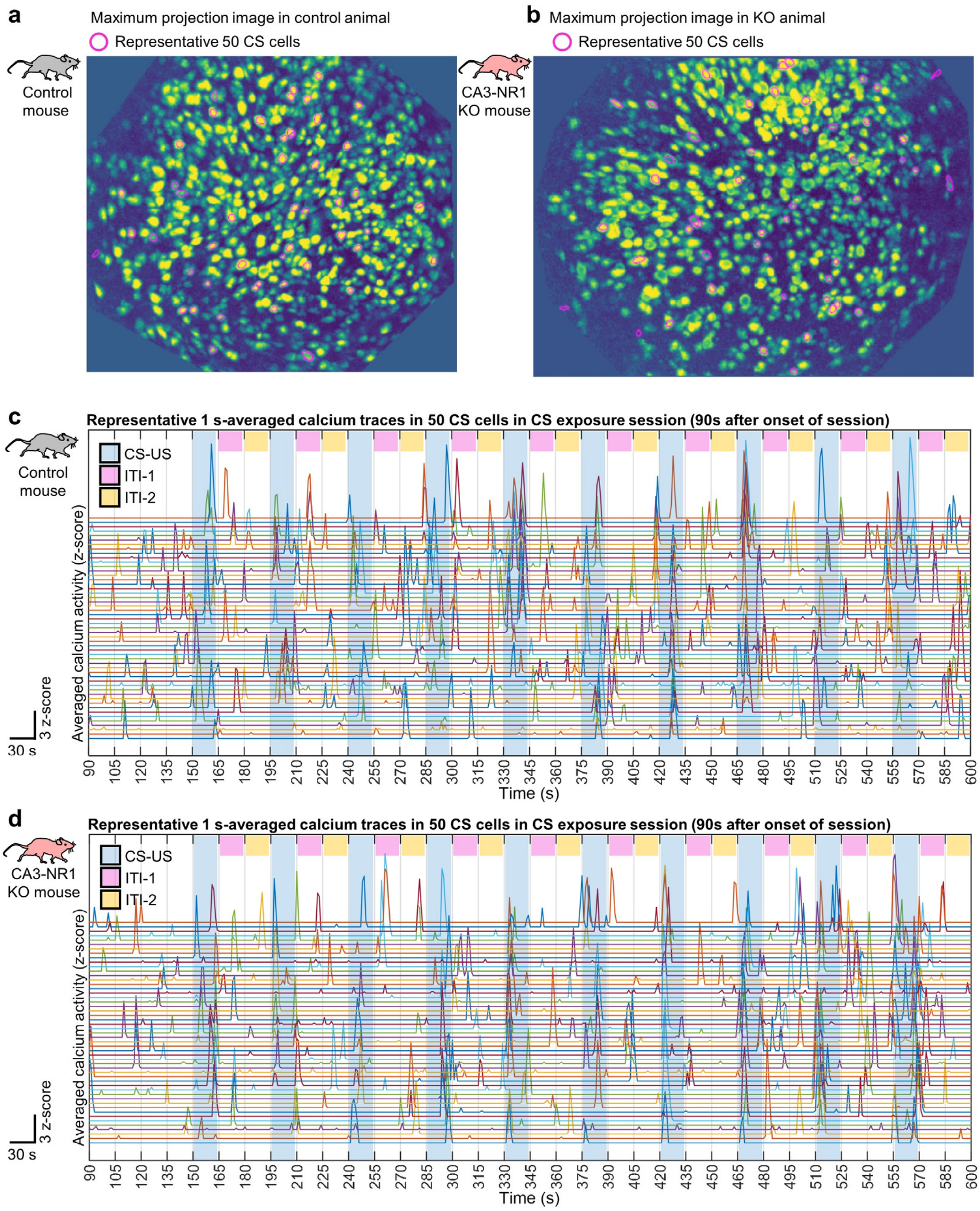

Supplementary Figure. 5 Representative *in vivo* calcium imaging data acquisition in CA1 of head-fixed animals

a, **b** Stacked- and pseudo-colored dF/F images acquired through the microendoscope over entire recording sessions of imaging in the hippocampus from (**a**) control and (**b**) KO animals. Magenta circles indicate the footprint contours of detected cells. **c**, **d** Representative 1 s-averaged calcium activities in 50 representative CS-responsive cells during LFC training (90 s after beginning the behavioral session to the end) in (**c**) control and (**d**) KO animals.

Blue, pink, and yellow rectangles indicate the timings of CS-US, ITI-1, and ITI-2, respectively.

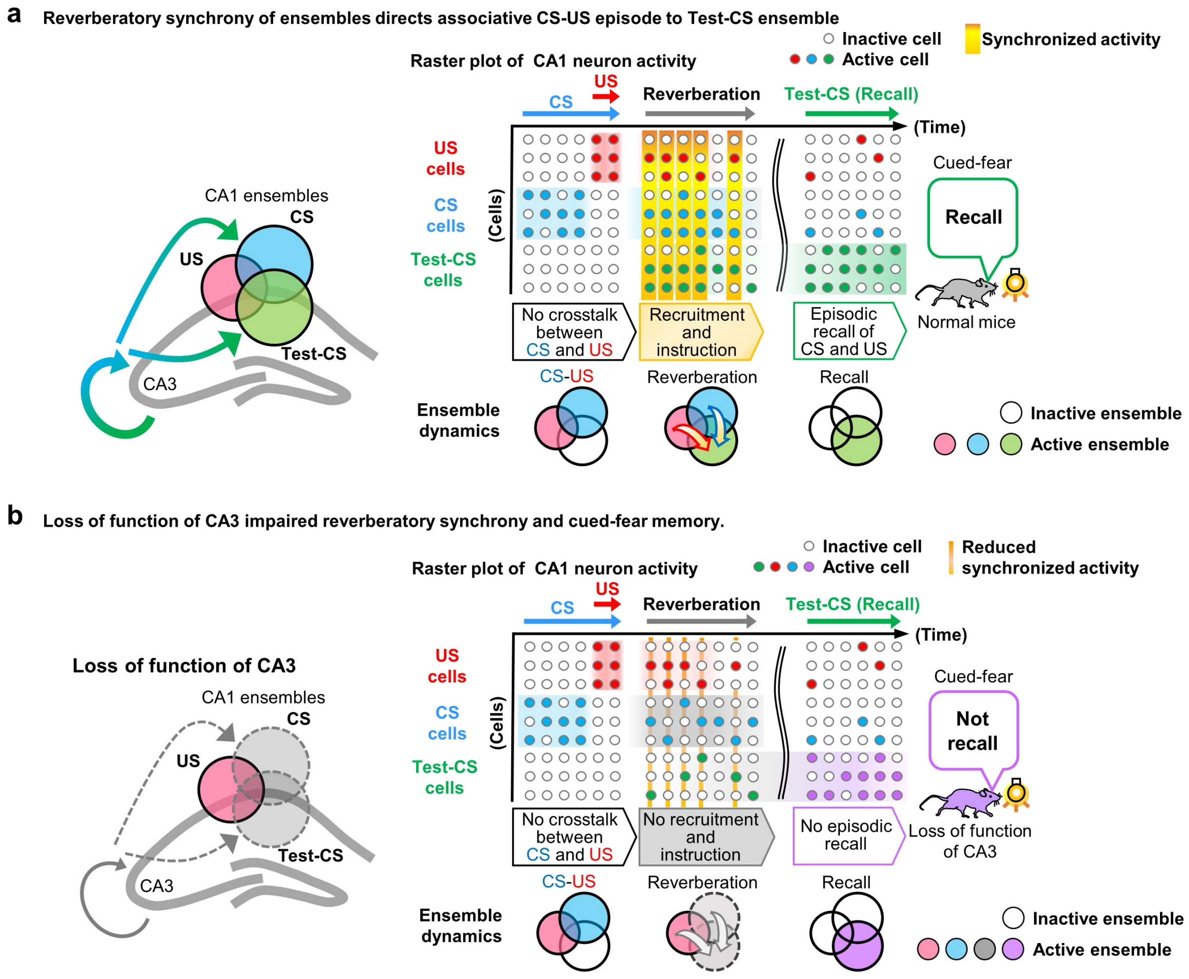

Supplementary Figure. 6 Model for cued-fear memory encoding in the hippocampal network using CA3-dependent reverberatory activity

a Left, Venn diagrams showing CS-, US-, and Test-CS-responsive cell ensembles in CA1 of normal mice, in which CA3-dependent reverberation occurs normally. Right, raster plots of CA1 subpopulations and with the timeline of the cued-fear memory paradigm. During CS and US inputs during training, CS and US information are separately encoded in CS- and US-responsive cell populations, respectively. During reverberation in training, co-activity of CS- and US-responsive cells recruits and instructs Test-CS-responsive cells in the CS-US episode. During recall, Test-CS-responsive cells drive the episodic recall of cued-fear memory. **b** Left, Venn diagrams of CS-, US-, and Test-CS-responsive ensembles in loss-of-function (CA3-NR1 KO and CA3 silencing during ITI). Right, during CS and US input in training, CS and US information are separately and normally encoded. However, without reverberation in

training, the low co-activity of CS- and US-responsive cells fails to recruit and instruct Test-CS-responsive cells in the episodic relation between CS and US. Thus, during recall, Test-CS-responsive cells fail to drive cued-fear memory recall. Note that synchrony rate in reverberation is comparable between genotypes. In contrast, synchrony index, which reflects the number of CS and Test-CS cells contributing to the synchrony events, is lower in CA3-NR1 KO mice. Filled circles with color indicate activated cells in each behavioral session. Arrows indicate the direction of information flow. Light bulb, light CS; yellow bar, moment-occurring synchrony among CS-, US-, and Test-CS cell ensembles.

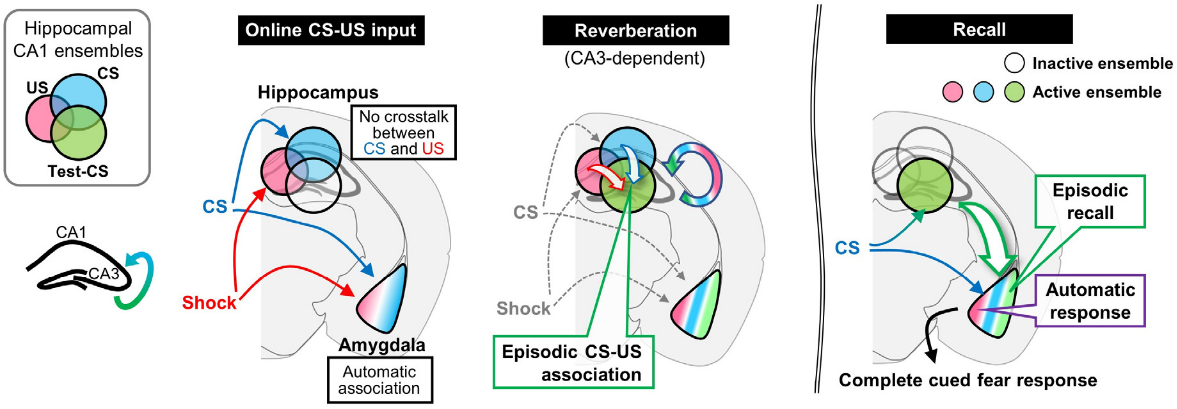

Supplementary Figure. 7 Model for hippocampal function in cued-fear memory

Venn diagrams showing CS-, US-, and Test-CS-responsive cell ensembles in CA1. During online CS and US inputs in training, the hippocampus encodes CS and US information independently, while the amygdala associates CS and US directly as automatic association. During reverberation, the hippocampus produces episodic CS-US association. During recall, the Test-CS-responsive cell ensemble in the hippocampus sends the episodic portion of the CS-US information to the amygdala to complete cued-fear memory. Filled circles and amygdala icons with color indicate temporal activation throughout learning and recall.
