# Supplementary material for: Hippocampus as a sorter and reverberatory integrator of sensory inputs
Source: Nat Commun. 2022 Dec 20;13:7413. doi: 10.1038/s41467-022-35119-2 (PMC9768143; doi:10.1038/s41467-022-35119-2)
Supplement: Supplementary file 3 — Description of Additional Supplementary Files [file 41467_2022_35119_MOESM3_ESM.pdf]

File name: Supplementary Data 1

Description: An Excel spreadsheet that contains details of the statistics.

File name: Supplementary Movie 1

Description: Calcium imaging demo movies in freely moving animals.

File name: Supplementary Movie 2

Description: Calcium imaging demo movies in head-fixed animals.
