# Supplementary material for: Hippocampus as a sorter and reverberatory integrator of sensory inputs
Source: Nat Commun. 2022 Dec 20;13:7413. doi: 10.1038/s41467-022-35119-2 (PMC9768143; doi:10.1038/s41467-022-35119-2)
Supplement: Supplementary file 7 — Reporting Summary [file 41467_2022_35119_MOESM7_ESM.pdf]

nature portfolio

Corresponding author(s): Kaoru Inokuchi

Last updated by author(s): Nov 12, 2022

Reporting Summary

Nature Portfolio wishes to improve the reproducibility of the work that we publish. This form provides structure for consistency and transparency in reporting. For further information on Nature Portfolio policies, see our Editorial Policies and the Editorial Policy Checklist.

Statistics

For all statistical analyses, confirm that the following items are present in the figure legend, table legend, main text, or Methods section.

n/a	Confirmed	
☐	☒	The exact sample size (*n*) for each experimental group/condition, given as a discrete number and unit of measurement
☐	☒	A statement on whether measurements were taken from distinct samples or whether the same sample was measured repeatedly
☐	☒	The statistical test(s) used AND whether they are one- or two-sided<br>*Only common tests should be described solely by name; describe more complex techniques in the Methods section.*
☐	☒	A description of all covariates tested
☐	☒	A description of any assumptions or corrections, such as tests of normality and adjustment for multiple comparisons
☐	☒	A full description of the statistical parameters including central tendency (e.g. means) or other basic estimates (e.g. regression coefficient) AND variation (e.g. standard deviation) or associated estimates of uncertainty (e.g. confidence intervals)
☐	☒	For null hypothesis testing, the test statistic (e.g. *F*, *t*, *r*) with confidence intervals, effect sizes, degrees of freedom and *P* value noted<br>*Give P values as exact values whenever suitable.*
☒	☐	For Bayesian analysis, information on the choice of priors and Markov chain Monte Carlo settings
☒	☐	For hierarchical and complex designs, identification of the appropriate level for tests and full reporting of outcomes
☐	☒	Estimates of effect sizes (e.g. Cohen's *d*, Pearson's *r*), indicating how they were calculated

Our web collection on statistics for biologists contains articles on many of the points above.

Software and code

Policy information about availability of computer code

Data collection	Freezing data were collected with video tracking system, CompACT VAS/DV (Muromachi Kikai, Japan). Behavioural data were collected with screen recorder softwares, Bandicam (Bandisoft, Korea) or AG-desktop recorder (T.Ishii, Japan). OpenEx software (RX8-2, Tucker Davis Technologies, USA) was used for synchronized recording between the freezing data and in vivo calcium imaging data and for the control of light pulse delivery in the optogenetic experiment. In vivo calcium imaging data were collected with nVista acquisition software (Inscopix, USA). Histlogical image acquisition from target region in optogenetic and imaging studies was performed with Keyence microscope (BIO-REVO, KEYENCE, Japan).
Data analysis	In vivo calcium imaging data were analyzed with Inscopix data processing software (IDPS, Inscopix, USA), Inscopix Mosaic software (Mosaic, Inscopix, USA), Fiji software (version 1.53q, NIH, USA), automatic sorting system, HOTARU (version 3.3.2, see method), and MATLAB 2020b (Mathworks, USA) with custom-made codes (https://github.com/IdlingBrainUT/Nomoto2022_NatureCommunications and https://doi.org/10.5281/zenodo.7293824). The statistical analysis was performed with Excel (Microsoft) with Statcel4 (OMS, Japan) and MATLAB 2020b (Mathworks, USA).

For manuscripts utilizing custom algorithms or software that are central to the research but not yet described in published literature, software must be made available to editors and reviewers. We strongly encourage code deposition in a community repository (e.g. GitHub). See the Nature Portfolio guidelines for submitting code & software for further information.

Data

Policy information about availability of data

All manuscripts must include a data availability statement. This statement should provide the following information, where applicable:

- Accession codes, unique identifiers, or web links for publicly available datasets
- A description of any restrictions on data availability
- For clinical datasets or third party data, please ensure that the statement adheres to our policy

The data are available from the corresponding author upon request. The datasets supporting this study will be deposited to a public repository when the ongoing studies using the same dataset are published. Source data are provided with this paper.

Field-specific reporting

Please select the one below that is the best fit for your research. If you are not sure, read the appropriate sections before making your selection.

☒ Life sciences ☐ Behavioural & social sciences ☐ Ecological, evolutionary & environmental sciences

For a reference copy of the document with all sections, see nature.com/documents/nr-reporting-summary-flat.pdf

Life sciences study design

All studies must disclose on these points even when the disclosure is negative.

Sample size	No statistical methods were used to predetermine sample sizes, which were based on work in previous publications (Ohkawa et al. Cell Reports 11, 261-269, 2015; Nomoto et al. Nature Communications 7, 12319, 2016; Yokose et al. Science 355, 398-403, 2017; Ghandour, K. et al Nature Communications 10, 2637, 2019).
Data exclusions	Mice in which poor viral expression, poor fluorescence image, an abnormal behavior, or teleopt cannula-malfunction were confirmed were excluded as described in the manuscript. Especially, mice showing an abnormal behavior or unclear fluorescent image were excluded prior to starting the experiments. In both freely moving and head-fixed imaging experiments, only completely motion-corrected data were used, and otherwise data with an inadequate frame or could not be corrected were excluded from analysis.
Replication	Independent animals were used for all studies as replicates. All experiments were repeated at least two times independently with similar results.
Randomization	Mice were randomly assigned to each groups, and experiments were run by alternating between each groups.
Blinding	All behavioural experiments were performed and analyzed by a researcher blind to experimental conditions with the exception of imaging experiment. Freezing data in optogenetic and imaging experiments were manually re-reanalyzed by other non-behavioural operators in blind condition with the same criteria to an automatic scoring, to exclude the effect of the attachment of optgenetic devices (optic fiber for wired optogenetics or Teleopt battery for wireless optogenetics), and calcium imaging device (cable and miniature microscopy nVista) on the automated animal tracking.

Reporting for specific materials, systems and methods

We require information from authors about some types of materials, experimental systems and methods used in many studies. Here, indicate whether each material, system or method listed is relevant to your study. If you are not sure if a list item applies to your research, read the appropriate section before selecting a response.

Materials & experimental systems

n/a	Involved in the study	
☐	☒	Antibodies
☒	☐	Eukaryotic cell lines
☒	☐	Palaeontology and archaeology
☐	☒	Animals and other organisms
☒	☐	Human research participants
☒	☐	Clinical data
☒	☐	Dual use research of concern

Methods

n/a	Involved in the study	
☒	☐	ChIP-seq
☒	☐	Flow cytometry
☒	☐	MRI-based neuroimaging

Antibodies

Antibodies used	Primary Antibodies: rabbit anti-GFP (1:500, A11122, Molecular Probes, USA), mouse anti-RGS-14 (1:500, N133/21, NeuroMab, USA), rabbit anti-DsRed (1:1000, 632496, Clonetech-Takara Bio, Japan).

	Secondary antibodies: donkey anti-rabbit IgG-AlexaFluor 488 (1:500, A21206, Molecular Probes, USA), donkey anti-mouse IgG-AlexaFluor 546 secondary antibodies (1:500, A11036, Molecular Probes, USA), goat anti-rabbit IgG-AlexaFluor 546 secondary antibodies (1:300, A11035, Molecular Probes, USA).
Validation	Each antibody is commercially available. The specificity of these antibodies was validated by the manufacturers.<br>Validation profiles for each antibody can be found in the links provided.<br><br>rabbit anti-GFP (1:500, A11122, Molecular Probes, USA: https://www.thermofisher.com/antibody/product/GFP-Antibody-Polyclonal/A-11122).<br>mouse anti-RGS-14 (1:500, N133/21, NeuroMab, USA: https://www.antibodiesinc.com/products/anti-rgs14-antibody-n133-21-75-170).<br>rabbit anti-DsRed (1:1000, 632496, Clonetech-Takara Bio, Japan: https://www.takarabio.com/products/antibodies-and-elisa/fluorescent-protein-antibodies/red-fluorescent-protein-antibodies?catalog=632496).<br><br>donkey anti-rabbit IgG-AlexaFluor 488 (1:500, A21206, Molecular Probes, USA: https://www.thermofisher.com/antibody/product/Donkey-anti-Rabbit-IgG-H-L-Highly-Cross-Adsorbed-Secondary-Antibody-Polyclonal/A-21206).<br>donkey anti-mouse IgG-AlexaFluor 546 secondary antibodies (1:500, A11036, Molecular Probes, USA: https://www.thermofisher.com/antibody/product/Donkey-anti-Mouse-IgG-H-L-Highly-Cross-Adsorbed-Secondary-Antibody-Polyclonal/A10036).<br>goat anti-rabbit IgG-AlexaFluor 546 secondary antibodies (1:300, A11035, Molecular Probes, USA: https://www.thermofisher.com/antibody/product/Goat-anti-Rabbit-IgG-H-L-Highly-Cross-Adsorbed-Secondary-Antibody-Polyclonal/A-11035).

Animals and other organisms

Policy information about studies involving animals; ARRIVE guidelines recommended for reporting animal research

Laboratory animals	Male CA3-NR1 KO mice, their floxed-NR1 littermates as control, and KA1::Cre were used for experiments.<br>KA1::Cre (C57BL/6-Tg(Grik4-cre)G32-4Stl/J, Stock No: 006474 | G32-4 Cre, Jackson Laboratory, USA: https://www.jax.org/strain/006474).<br>Floxed-NR1 (B6.129S4-Grin1tm2Stl/J, Stock No: 005246 | fNR1 ,Jackson Laboratory stock no: 005246) which backcrossed with C57BL/6J more than 25 times were donated by Drs. S. Tonegawa [RIKEN-Massachusetts Institute of Technology] and S. Itohara [RIKEN Brain Science Institute]: https://www.jax.org/strain/005246).<br>All mice were maintained on a 12 h light/dark cycle (lights on 8:00 am – 8:00 pm) at 24 ± 3°C and 55 ± 5% humidity, had ad libitum access to food and water, and were housed in a cage with littermates until surgery. Mice were 16-26 weeks old.
Wild animals	This study did not involve wild animals.
Field-collected samples	This study did not involve samples collected from the field.
Ethics oversight	All animal procedures were approved by the Animal Care and Use Committee of the University of Toyama (Approval numbers: A2019MED-35, A2022MED-7).

Note that full information on the approval of the study protocol must also be provided in the manuscript.
